# Centering Diverse Communities within Mindful Parenting Interventions in the U.S.: A Narrative Literature Review

**DOI:** 10.3390/ijerph21101360

**Published:** 2024-10-15

**Authors:** J. Corey Williams, Noel Bravo, Preeti Kota, Janaire Hawkins, Katherine Greene

**Affiliations:** 1 Early Childhood Innovation Network, Washington, DC 20007, USA; 2 MedStar Georgetown University Hospital, Washington, DC 20007, USA; 3 Department of Pediatrics, Georgetown University School of Medicine, Washington, DC 20007, USA; 4 College of Arts and Sciences, Georgetown University, Washington, DC 20057, USA; 5 Dahlgren Memorial Library, Georgetown University, Washington, DC 20057, USA

**Keywords:** mindfulness, parenting, diversity

## Abstract

Background: While mindfulness-based interventions targeted toward parents (and families) in the U.S. offer promise for the treatment and prevention of youth psychological disorders, current research has established the underrepresentation of diverse participants in the research literature. The full extent of inequalities in the demographics of participation in parent mindfulness intervention is less understood. Objective: This study aimed to utilize a narrative literature review approach to examine and describe the degree to which research on mindful parenting interventions is inclusive of BIPOC (Black, Indigenous, and People of Color) communities, non-clinical samples (no diagnosed disorder), cultural adaptions, and skills specific to parenting. Methods: An electronic database search of US-based studies was undertaken for empirical studies that primarily focused on parent mindfulness interventions, which reported outcomes related to either parenting behaviors or child mental health outcomes. After a full-text review, the search resulted in 34 articles. A narrative literature review of the 34 studies was conducted to assess the inclusion of BIPOC communities, non-clinical samples, cultural adaptions, and skills specific to parenting. Results: This review found notable gaps in the degree to which mindful parenting research (1) included BIPOC populations in study samples; (2) focused on non-clinical samples; (3) adapted interventions to align with the cultural needs of participants; and (4) included the application of mindfulness to enhancing knowledge, skills, and behaviors specific to parenting. Conclusions: Given these gaps in the parent mindfulness literature, greater research attention is needed on mindful parenting interventions targeted toward BIPOC communities with no clinical diagnoses, interventions optimized by cultural adaptations, and explicit applications to parenting.

## 1. Introduction

The prevalence of mental health disorders among children and adolescents has dramatically risen over the last decade, with mental health disorders occurring among 16–20% of children ages 4 to 11, 20–25% of adolescents ages 12 to 17, and 25–40% of young adults ages 18 to 24 [1]. To address this rising demand for mental health support, there is an urgent need for the expansion of preventative family-centered interventions that can prevent or attenuate emergent mental health issues before diagnoses and clinical interventions are required. Mindfulness—commonly defined as non-judgmental awareness of one’s internal states and surroundings [2]—is receiving increased attention as an impactful intervention modality with treatment and preventative implications. Specifically, mindfulness-based interventions that target parents (i.e., mindful parenting interventions) are a well-researched approach that has the potential to benefit whole families [3].

The common objective of mindful parenting interventions is to promote the practices of present-moment, nonjudgmental awareness, such that parents enhance their stress management skills and emotional regulation in the context of parenting. The structure of these interventions typically includes didactic instruction, in-session exercises, and/or home-based exercises. Mindful parenting interventions can vary in mode of delivery (e.g., mobile app, in-person, etc.), frequency, duration, criteria for participation (e.g., parents only or parents and children), and the degree to which curriculum focuses on the application of parenting behaviors (e.g., disciplining children) [3].

In clinical populations (i.e., participants who have an identified medical or mental health condition), parent mindfulness interventions have shown significant benefits to both parents and children across a range of clinical diagnoses. For example, in parents diagnosed with depression, anxiety, post-traumatic stress disorder, and eating disorders, mindfulness-based interventions have been found to be comparable to standard treatments [4], and mindfulness interventions have been shown to significantly improve parents’ well-being and reduce children’s symptoms of ADHD [5].

However, little evidence exists for the potential benefit of mindful parenting interventions for non-clinical samples (i.e., parents or children without an identified diagnosis). The limited research that does exist in non-clinical samples has shown an association between parent participation in mindful parenting interventions with reduced stress and increased emotion regulation ability for parents [6], both of which contribute significantly to positive parenting behaviors and child development; however, more research is needed to fully evaluate the preventive benefits of mindful parenting interventions, especially for child developmental outcomes.

In addition to the dearth of literature on non-clinical samples, research on parent mindfulness (and mindfulness interventions broadly) has demonstrated a significant underrepresentation of BIPOC (Black, Indigenous, People of Color) participants within sample populations. A review of US-based randomized control trials of mindfulness-based interventions, in general, included 69 studies, of which only one specifically focused on racial/ethnic minority or lower socioeconomic status populations; 13 studies reported no data on race/ethnicity; 11 studies reported only the proportion of White participants; and no studies reported intervention effectiveness for disaggregated racial/ethnic groups [7]. These studies demonstrate that limited research focuses on understanding and expanding the benefits of mindfulness-based interventions for BIPOC communities. 

This lack of focus on BIPOC research participants is especially alarming in the context of current evidence suggesting that certain aspects of predominant Western mindfulness teaching are not culturally relevant for certain BIPOC communities [8]. The underrepresentation of BIPOC participation also represents not only how the effectiveness of mindfulness in BIPOC communities is understudied but also highlights the opportunities to optimize the efficacy of mindfulness by adapting interventions to the unique cultural, traditional, and spiritual needs of BIPOC communities [9]. While some researchers may be concerned that modifications to treatment manuals could compromise intervention fidelity, the literature for other manualized treatment modalities asserts that cultural adaptions—such as linguistic or conceptual alterations—enhance the adherence and effectiveness of an intervention [10], suggesting a similar approach could enhance parent mindfulness intervention in BIPOC families.

To address this gap in the literature, researchers need an enhanced understanding of how elements of mindfulness instruction can be adapted to optimize the cultural relevance of a given community context, especially BIPOC communities. This paper aims to qualitatively review the recent literature on parent mindfulness interventions, with a specific focus on non-clinical samples of BIPOC parents and the cultural adaptations within the intervention curriculum. Our goal with this narrative literature review was to better understand four main questions within the parent mindfulness intervention literature: to what extent do recent, U.S.-based studies report (1) the inclusion of BIPOC participants; (2) the inclusion of non-clinical samples; (3) adaptations of curriculum to the cultural context of participants; and (4) interventions that focus on developing specific parenting knowledge, skills, or behaviors? A narrative literature review was selected as the method of review to allow for a descriptive perspective, critical analysis, and synthesis of the current state of the literature focusing on these four key questions.

## 2. Materials and Methods

### 2.1. Search Strategy

An electronic search for English-language articles was conducted using the Ovid MEDLINE database. While a narrative literature review does not require the strict search and vetting process of a systematic review [11], our team opted for a systematic review approach to thoroughly capture the available published research. While systematic reviews routinely utilize study quality assessments, especially for reviews focused on clinical evidence, we opted not to use a specific quality assessment, as intervention effectiveness was not the focus of our narrative review. We restricted our search to contemporary literature from the last twenty years (2003–2023). Search terms included combinations of the following terms found anywhere within the record, both as Medical Subject Headings (MeSH) terms and keywords including “mindfulness”, “mindful”, “parents” and “children” (see Appendix A for Concept Table with search terms).

### 2.2. Screening Criteria

We included only intervention studies that reported empirical data; therefore, we excluded editorials, commentaries, perspective essays, case reports, and all reviews. Our inclusion criteria were further narrowed to include only studies that reported parenting-related outcomes and/or developmental outcomes for children, thereby excluding implementation and quality improvement studies that focused on program operations. Of note, we also reviewed the references within relevant systematic reviews and meta-analyses; however, we ultimately excluded all reviews during the screening process.

In addition, our screening criteria distinguished between mindfulness-based interventions (e.g., MBSR—Mindfulness-Based Stress Reduction) and mindfulness-informed interventions (e.g., acceptance and commitment therapy). Mindfulness-informed interventions often incorporate mindfulness techniques into existing mental health interventions but do not focus primarily on mindfulness, so we removed all studies utilizing mindfulness-informed intervention. This distinction between mindfulness-informed and mindfulness-based approaches has been used in prior literature [12]. Finally, we excluded studies conducted outside of the U.S. Although a wealth of research has been conducted in other countries, the current study focuses on the racial and cultural composition of the U.S. and does not presume that this context could be applied appropriately to the cultural contexts of other countries.

### 2.3. Study Selection Process

**Figure 1:**
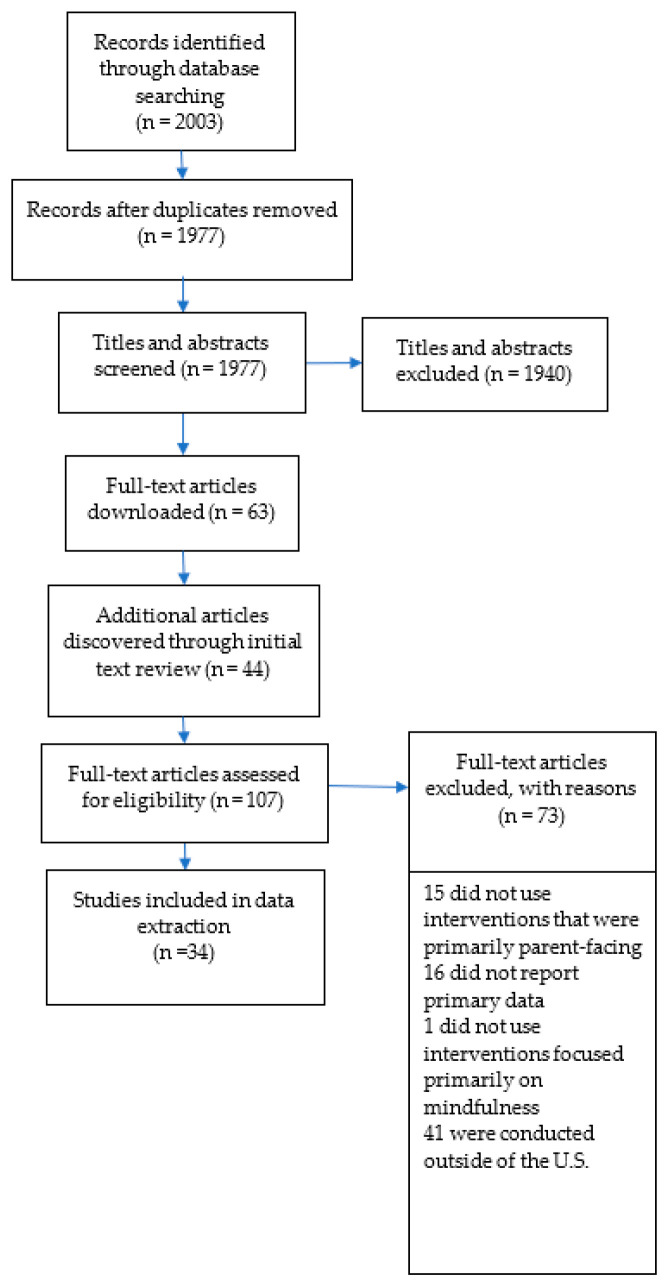
Workflow of the article review process.

Three independent reviewers (NB, JW, PK) participated in study selection in three general stages: (1) a title review, (2) an abstract review, and (3) a full-text review. The title and abstract reviews were conducted using Rayyan 1.5.0, a systematic review software. All titles assessed as relevant by at least one reviewer were included in the abstract review stages, and all abstracts deemed relevant were included in the full-text review. We utilized team discussions and team consensus to reconcile any disagreement between the three reviewers. At the full-text review stage, only those studies considered relevant by all three reviewers were selected and included for data extraction. During this stage, additional studies were identified by reference reviews of meta-analyses (and systematic reviews) for inclusion in the full-text review (see Figure 1 for flowchart).

### 2.4. Data Extraction

During the data extraction process, we focused on the degree of inclusion of BIPOC parents by extracting the racial and ethnic demographics that were reported in each study. Next, we extracted data relevant to any non-clinical samples, which we defined as parents/children who are typically developed or parents/children who did not have any identified medical, mental health, or other socioeconomic issues (e.g., homelessness). We also extracted any cultural adaptations reported in the design or delivery of the parent mindfulness interventions. We operationalized cultural adaptation to mean any modifications to the intervention curriculum based on the cultural, social, or historical context of the study population (such as modifications based on linguistic preferences, cultural heritage, spirituality, social needs, cultural values/priorities, etc.). Given this review’s focus on parenting competencies, we extracted data on whether studies applied mindfulness techniques to specific parenting knowledge, skills, and attitudes as well as parent–child interactions (e.g., disciplining children or enhancing parent–child attachment).

## 3. Results

### 3.1. General Study Characteristics

A total of 34 articles were identified as being eligible for data extraction for this narrative literature review. Table 1 presents the data extraction from all of the identified studies. The extracted studies were primarily randomized control trials (*n* = 12, 35% of studies), other quantitative methods (*n* = 12, 35% of studies), qualitative studies (*n* = 4, 12%), mixed methods studies (*n* = 3, 9%) and feasibility studies with preliminary outcome data (*n* = 3, 9%). Two studies were published as dissertation defenses [13,14]. The sample size of participating parents ranged from 7 to 160. For each included study, reviewers assessed the key areas of interest of the current review, as described below.

### 3.2. To What Extent Do Recent Studies Include BIPOC Participants?

A review of the data extracted from each study revealed that all but one study reported at least some data on the race/ethnicity of participants. Twenty studies (59%) reported that the majority of participants identified as White. In terms of reported racial/ethnic demographic data, in 11 studies (32%), the authors presented only the percentage of participants who were White. For instance, Chaplin et al. (2021b) [21] and Duncan et al. (2009) [24] reported that 65% of participants were White and 93% were White, respectively, without reference to the race/ethnicity of the other participants. Fifteen studies (44%) presented data indicating some participation from Black people. For Asian/Pacific Islander participants, 11 studies (32%) presented disaggregated data. Four studies (12%) reported data for American Indian participants. Sixteen studies (47%) reported data for Latine (a gender-neutral term that refers to people of Latin American heritage) participants. Most studies did not report the method by which researchers collected data on race/ethnicity; therefore, it is unknown whether these data were self-reported by participants, assigned by researchers, or based on other data sources.

### 3.3. To What Extent Do Recent Studies Focus on Non-Clinical Samples?

Six studies (18%) offered a mindful parenting intervention to a non-clinical community sample. The majority, 28 studies (82%), required participating parents or children participants to exhibit clinical symptoms, a diagnosed psychological disorder, or a socioeconomic issue (i.e., poverty/homelessness). Among these problem- or symptom-focused studies, 14 studies (41%) used a diagnosed disorder for the child (i.e., ADHD, ASD, or IDD) as the inclusion criterion. Seven studies (21%) focused on parental symptoms or disorders, consisting of parental stress, anxiety, or SUD/OUD. A smaller subset of studies (*n* = 3, 9%) focused on parents of children with medical issues requiring hospitalization (i.e., Neonatal Intensive Care Unit). Two studies (6%) focused on parents with physical challenges related to obesity or a genetic condition (i.e., FMR1 premutation) [31,32]. Three studies (9%) focused on parents with socioeconomic challenges of divorce, poverty, and/or homelessness [15,35,46].

### 3.4. To What Extent Do Recent Studies Include Adaptations of Curriculum According to the Cultural Context of Participants?

No studies reported developing or adapting its intervention to be aligned with the culture of participants. However, a few references were made to adapting the mode of delivery, duration, and frequency of mindful parenting activities to the availability of participants (e.g., busy parents or antepartum women). For example, Doty et al. (2022) [23] utilized a smartphone-based, self-guided mindfulness intervention because it required minimal training and provided maximum flexibility of use, given the attentional demands women experience in an antepartum hospital setting. Another study [24] offered childcare, dinner, and attendance gifts to parents to facilitate participation. Anderson et al. (2015) [16] adapted content to reference the unique life challenges of a child’s psychopathology and discussed how the intervention included applications to the specific challenges of caring for a child with ADHD. No study reported making linguistic alterations according to participants; no studies cited adaptions to the curriculum according to the cultural values and priorities of the participants; and no studies reported any consideration of racial/ethnic concordance between facilitators and participants.

### 3.5. To What Extent Do Studies Focus on Applying Mindfulness to Knowledge, Skills, and Behaviors Specific to Parenting?

All studies taught core mindfulness practices in some form, such as deep breathing, body awareness, meditation, present-moment awareness, and body scan exercises. Twelve studies (35%) focused solely on the practice of mindfulness for the individual participants and offered no specific or intentional reference to how this practice applies to parenting knowledge, skills, or behaviors. Twenty-two studies (65%) explicitly included applications to parenting in their intervention description. For example, several studies included an explicit focus on the mindful parenting capacities of listening with full attention; emotional awareness of, acceptance of, and compassion for self and child; and self-regulation in parenting [22,24,43]. One intervention went so far as to engage with parent/child dyads in mindful and intentional play while providing real-time feedback on parenting behaviors [28].

## 4. Discussion

This narrative literature review sought to describe the U.S. landscape of parent mindfulness research with a specific focus on the participation of BIPOC, intervention adaptions based on culture or parenting, and non-clinical samples. In summary, several gaps in the literature became apparent from this narrative literature review. Overall, we found a significant underrepresentation of both BIPOC and non-clinical samples, a lack of research on cultural adaptations of curriculum, and a dearth of studies that specifically employ mindfulness curricula that target parenting-related competencies.

With regards to sample representative diversity, over half of studies reported that a majority of participants identified as White, and almost a third of studies reported participation data for White participants only. Several factors may be driving a sampling and reporting phenomena that favor White-identifying participants. For one, prior research has shown that many intervention studies take place in hospitals, clinics, or university settings that have existing racial disparities in access to services [47]; thus, potential participant pools are limited to predominantly White samples. Plus, traditional non-participatory research designs (e.g., RCTs) typically do not involve significant community outreach efforts to engage participants who have been historically excluded from intervention studies [48]. Further, the fact that only 18% offered a mindful parenting intervention to a non-clinical community sample indicates that mindful parenting interventions are primarily researched as clinical interventions in response to diagnosed disorders rather than a public health approach to the prevention of illness or promotion of healthy child development and family functioning.

This narrative literature review did not identify any studies that developed or adapted an intervention in alignment with the cultural identity of the participants. We speculate that this gap in the literature may represent researchers’ tendency to prioritize standardized training content in the interest of preserving intervention fidelity. Further, this gap may reflect a lack of consideration among researchers that participant cultural identity can play an important role in the acceptance, adherence, and efficacy of the mindfulness intervention. In the broader psychosocial intervention literature, interventions like Cognitive Behavioral Therapy (CBT) have shown robust results with cultural adaptations, including linguistic modifications [10]. Also, current evidence suggests that the culture of study participants—specifically BIPOC parents—plays an important role in the feasibility and acceptability of mindful parenting interventions [8,49], and the bias embedded in standardized research protocols (designed to advance generalizable interventions) can come at the cost of cultural and local specificity.

Cultural adaption can present implementation challenges for researchers and practitioners, especially given that cultural traditions within a population are heterogeneous and fluid. Guidance for navigating these challenges can be drawn from a wealth of literature on participatory research approaches, such as community-based participatory research (CBPR). CBPR approaches have demonstrated how interventions can be culturally adapted while maintaining adequate fidelity and enhancing intervention efficacy [50]. Plus, this team has published data elsewhere [49,51,52], suggesting how CBPR in the context of mindfulness interventions can lead to cultural adaption and innovations in curriculum, outcome measures, and data interpretation.

The fact that only approximately two-thirds of the studies included explicit applications to parenting in their intervention curriculum raises an important question regarding the design of mindful parenting interventions. While decades of empirical scholarship have built a strong case for developing individual mindfulness practices that promote general wellness among parents, which in turn promote positive parenting [4], explicit training on applying mindfulness concepts and practices to parenting behaviors is under-studied. Emphasizing specific applications of mindfulness to parenting can potentially enhance the impact of mindful parenting training on parent–child interactions, thus, fostering healthy child development. Future studies should explore how explicit application to parenting through activities, such as applying present-moment awareness to interactions with children, or during play, can improve child development and other metrics of family well-being.

Several limitations emerged throughout this review, which may have impacted the findings. Notably, the specificity of our aims required the exclusion of many studies related to mindful parenting that may have offered additional observations. The exclusion of international studies did not allow for the examination of research in non-U.S. contexts, and the focus on mindfulness-based (vs. mindfulness-inspired) interventions led to the exclusion of some parent-involved research related to mindfulness that could have yielded additional insights. Finally, the majority of the selected studies did not include detailed descriptions of the mindfulness curriculum that was utilized, which may have contributed to an under-recognition of specific cultural adaptations or parenting applications in the interventions, but were not fully described within published articles.

## 5. Conclusions

Mindful parenting interventions offer great promise as a whole-family and preventative approach to ameliorating emergent youth mental health challenges. This review has revealed that both the potential preventative benefits and the benefits within parent–child interactions for mindful parenting interventions are understudied areas. These interventions need not be restricted to clinically treated populations, and they can be made more broadly available to families through a public health approach, serving families in educational, recreational, religious, and broader community settings. And, to ensure BIPOC communities benefit from these interventions, cultural adaptations, and targeted community engagement strategies should be developed, implemented, and evaluated.

## Figures and Tables

**Table 1:** Data extraction.

Author/Year	Study Design	Inclusion of BIPOC Parents	Clinical vs. Non-Clinical Samples ^1^	Cultural Adaptations ^2^	Parenting Knowledge, Skills, or Behavioral Outcomes
Alhusen et al., 2017 [15]	Qualitative study assessing benefits of *Mindful Awareness Play* and *Support, Honor, Inspire, Nurture, Evolve* for mothers (*n* = 17) and their children	71% of mothers were Black, 29% White	Clinical—Mothers/children experiencing homelessness	None reported	Curious exploration and nonjudgmental play with child, interactive patterns that promote attachment relationship, mutual regulation, and address trauma-induced developmental delays
Anderson et al., 2015 [16]	Pre-post assessment of *Everyday Blessings: The Inner Work of Mindful Parenting* intervention impact on stress for mothers (*n* = 7)	57% of mothers were White, 29% Asian, 14% Other	Clinical—Parents of children with ADHD ^3^	None reported	Bringing awareness and intentionality to parenting practices
Bazzano et al., 2013 [17]	Pre-post assessment of *Mindfulness-Based Stress Reduction* on stress and parental stress for parents (*n* = 59) and caregivers (*n* = 7)	Parents/caregivers were 45% Latine, 32% White, 12% Black, 11% other	Clinical—Parents of children with DD ^4^	Community members participated in design of intervention and study through a CBPR ^5^ approach	None—Focused on individual mindfulness practice
Burke et al., 2017 [18]	Qualitative study of experience with modified *Mindfulness-Based Stress Reduction* among parents and teachers (*n* = 26)	46% of participants were White, 4% Black, 35% Latine, 8% Asian, 8% other	Clinical—Parents of children with IDD ^6^	None reported	None—Focused on individual mindfulness practices
Chan et al., 2018 [19]	RCT ^7^ of *Mindfulness-Based Stress Reduction* impact on parenting behaviors, parenting stress, and child emotion dysregulation for parent/child dyads in immediate treatment (*n* = 41) and waitlist control (*n* = 39) groups	Parents were 48% Latine, 25% White, 21% other, 4% Asian, 3% Black	Clinical—Parents of children with DD	None reported	Sensitive parenting and intrusive parenting
Chaplin et al., 2021a [20]	RCT assessing impact of *Parenting Mindfully* on parenting and parent stress with parent-adolescent dyads in treatment (*n* = 41) and control (*n* = 42) groups	65% of adolescents were non-Latine White	Clinical—Parents with high-stress	None reported	Awareness in interactions and emotions, acceptance of self and child, non-reactivity to child behaviors, compassion for child
Chaplin et al., 2021b [21]	RCT assessing impact of *Parenting Mindfully* on adolescent SUD and psychopathology with mother-adolescent dyads in treatment (*n* = 48) and control (*n* = 48) groups	65% of adolescents were White	Clinical—Mothers with high stress	None reported	Awareness in interactions and emotions, acceptance of self and child, non-reactivity to child behaviors, compassion for child
Coatsworth et al., 2015 [22]	Randomized control comparative effectiveness study assessing impact of *Mindfulness-Enhanced Strengthening Families Program* on parenting with parents in treatment (*n* = 23) and control (*n* = 23) groups and on behavior problems of their children	69% of parents were White, 15% African American, 8% Latine, 4% Asian, 1% American Indian, 3% biracial	Non-clinical—Open recruitment through public school system	None reported	Listening with full attention. Emotional awareness of, acceptance of, and compassion for self/child. Self-regulation in parenting.
Doty et al., 2022 [23]	RCT assessing impact of *Mindfulness-Based Stress Reduction* and *Calm* on antepartum anxiety, stress and depression with antepartum mothers in treatment (*n* = 28) and control (*n* = 28) groups	27% of parents White, 45% Black, 25% Latine, 4% other	Non-clinical—Antepartum women	None reported	None—focused on standard individual mindfulness practices of *MBSR* and *Calm* app use
Duncan et al., 2009 [24]	Mixed-method acceptability evaluation of adapted *Strengthening Families Program* for parents (*n* = 9)	93% of parents were White	Non-clinical—Open recruitment through public school system	None reported	Listening with full attention. Emotional awareness of, acceptance of, and compassion for self/child. Self-regulation in parenting.
Felver et al., 2014 [25]	RCT assessing impact of *Mindful Family Stress Reduction* on children’s attentional regulation with parent-infant dyads in treatment (*n* = 24) and control (*n* = 23) groups	98% of families were White	Non-clinical—Open recruitment	None reported	Interpersonal communication skills
Ferraioli et al., 2012 [26]	Comparison of mindfulness-based and skills-based parent training impact on parental stress and global health outcomes for parents (*n* = 15)	Parents were 33% White, 27% Indian, 13% Asian, 13% Latina, 7% Black, 7% other	Clinical—Parents of children with ASD ^8^	None reported	Incorporation of mindfulness skills (observing, describing events and personal responses, nonjudgmental acceptance, distancing from thoughts, staying present, and being effective) into daily life
Gannon et al., 2017 [27]	Mixed methods study assessing impact of *Mindfulness-Based Parenting* on mindful/interacting parenting by mothers (*n* = 160)	76% of mothers were White, 7% Black, 8% multiracial, 9% Latine	Clinical—Mothers with OUD ^9^	None reported	Impact of trauma on parenting, listening with full attention, nonjudgmental acceptance, emotional awareness of self and child, self-regulation, and compassion for self and child
Gannon et al., 2022 [28]	Qualitative assessment of how *Mindfulness-Based Parenting* group affected mindfulness in daily lives and relationships with children of mothers (*n* = 40)	68% of mothers were White and 77% non-Latine	Clinical—Mothers post-partum in SUD ^10^ treatment	None reported	Mindful and intentional play with real-time feedback on parenting behaviors
Goodman et al., 2014 [29]	Pre- to post-comparison study assessing impact of *CALM Pregnancy* on anxiety of pregnant women (*n* = 23)	75% of women were White/non-Latine, 13% Asian, 8% Latine, 4% other	Clinical—Pregnant women with anxiety	None reported	Using mindfulness in pregnancy, labor and delivery, and parenting
Guenther et al., 2021 [30]	Feasibility/acceptability study of *Parent–Child Mindfulness-Based Training* on child mindfulness and memory with parent–child dyads in treatment (*n* = 14) and control (*n* = 8) groups	95% of participants were White	Non-clinical —Healthy children	None reported	None—focused on individual mindfulness practices (e.g., mindful eating and walking, noticing thoughts and feelings)
Hunter et al., 2019 [31]	Pre- to post-feasibility study assessing impact of *Headspace Take 10* on stress and social anxiety with mothers (*n* = 18)	83% of mothers were White	Clinical—Women who carry FMR1 ^11^ premutation and have children with fragile X syndrome	None reported	None—focused on individual mindfulness practices
Jastreboff et al., 2018 [32]	RCT assessing impact of *Parenting Mindfully for Health* on parenting and child obesity with parent–child dyads in treatment (*n* = 19) and control (*n* = 15) groups	Participants were 37–38% White	Clinical—Parents with obesity	None reported	Mindful parenting concepts, mindful eating with family, awareness of child’s body and family lifestyle choices
Kantrowitz-Gordon et al., 2018 [33]	Qualitative study of experience with *Mindfulness for Childbirth and Parenting* among pregnant women (*n* = 12)	100% of mothers were White	Clinical—Pregnant women	None reported	Being present with baby, promoting loving kindness toward baby
Lewallen et al., 2015 [34]	Pre-to post-assessment of *Mindfulness-Based Stress Reduction* impact on parental stress, parent–child relational factors, and child social skills for families (*n* = 21)	Children were 38% Latine, 33% White, 21% Other, and 8% Asian	Clinical—Parents of children with DD	None reported	Discipline practices, communication, and involvement
Maloney et al., 2007 [35]	Pre- to post-assessment of *Mindful Parenting Program* impact on parental mindfulness and parent–child interactions for parent/child dyads (*n* = 12)	Parents were 100% White	Non-clinical—Divorced or permanently separated parents	None reported	Dyadic process (positive engagement, mutual warmth, happy emotional tone, reciprocity, and mutual intimacy of topic) and parent behavior (responsiveness, reflecting, and validation)
Marshall et al., 2019 [36]	Pre-to post-assessment of *Mindfulness-Based Training Session* impact on stress and anxiety for parents (*n* = 28)	69% of parents White, 22% Black, 9% unreported	Clinical—Parents of preterm neonates in NICU ^12^	None reported	None—focused on individual mindfulness practices
Mendelson et al., 2018 [37]	Mixed methods study assessing impact of *novel mindfulness intervention* on stress with mothers (*n* = 24)	Mothers were 54% White, 42% Black, 4% Asian/PI, 13% Latine	Clinical—Parents of infants in NICU	None reported	None—focused on individual mindfulness practice practices
Neece et al., 2014 [38]	RCT assessing impact of *Mindfulness-Based Stress Reduction* on stress, depression, and life satisfaction with parents in treatment (*n* = 23) and control (*n* = 23) groups and on behavior problems of their children	26% of parents were White, 37% Latine, 9% Asian, 6% Black, and 23% other	Clinical—Parents of children with developmental delays	None reported	None—focused on individual mindfulness practices
Noroña-Zhou et al., 2022 [39]	RCT assessing impact of *Mindfulness-Based Childbirth and Parenting* on infant reactivity/regulation with mother-infant dyads in treatment (*n* = 71) and control (*n* = 63) groups	17% of infants were White, 36% Black, 1% Asian, 1% Native American, 46% mixed race or other, 40% Latine	Clinical—Mothers with high stress	None reported	None—focused on individual mindfulness practice (e.g., eating behavior, mindful eating, and stress reduction)
Petteys et al., 2018 [40]	RCT assessing impact of *mindfulness-based neurodevelopmental care* on anxiety with parent/infant dyads mothers in treatment (*n* = 27) and control (*n* = 28) groups	Among subset of parents (*n* = 44), 61% were Latine	Clinical—Parents of preterm infants in NICU	None reported	Awareness and nonjudgment of infant, attunement, touch, observation and recognition of infant cues
Roberts et al., 2015 [41]	Feasibility assessment of delivering *Mindfulness-Based Stress Reduction* to parents in treatment (*n* = 18) and control (*n* = 25) groups	63% of parents were minority, of which 40% were Latine	Clinical—Parents of children with DD	None reported	Focused on individual mindfulness practices and incorporating mindfulness into everyday life to facilitate more adaptive responses to challenges
Roberts et al., 2020 [42]	Pre- to post-assessment of *Mindfulness-Based Stress Reduction* impact on stress and cortisol awakening response for parents (*n* = 47)	No data on race/ethnicity reported, although some participants spoke Spanish	Clinical—Parents of children with DD	None reported	None—focused on individual mindfulness practices
Short et al., 2017 [43]	Pre- to post-assessment of *Mindfulness-Based Parenting* impact on stress for mothers (*n* = 59)	73% of mothers were White, 5% black, 5% multiracial, 14% other, 2% unknown	Clinical—Mothers with OUD	None reported	Listening with full attention. Emotional awareness of, acceptance of, and compassion for self/child. Self-regulation in parenting.
Voos, 2017 [13]	Repeated measures assessment of *Mindful Parenting* impact on mindfulness, parenting stress, and parent–child relationship quality for parent/child dyads (*n* = 21)	Parents were 86% White, 10% Latine, 10% mixed, 5% Native Hawaiian	Clinical—Parents of children with ASD	None reported	Parenting stress and parent–child relationship quality
Weitlauf et al., 2020 [44]	RCT assessing impact of *Mindfulness-Based Stress Reduction* on stress with parent/child dyads in treatment (*n* = 30) and control (*n* = 31) groups	Majority of parents were White	Clinical—Parents of children with ASD	None reported	None—focused on individual mindfulness practices
Weitlauf et al., 2022 [45]	RCT assessing impact of *Mindfulness-Based Stress Reduction* on child functioning with parent/child dyads in treatment (*n* = 30) and control (*n* = 32) groups	Children were 84% White, 6% Asian, 3% Black, 3% Native American, 3% other	Clinical—Parents of children with ASD	None reported	None—focused on individual mindfulness practices
Xu, 2017 [14]	Pre- to post-assessment of *Mindfulness-Based Stress Reduction* impact on parental meta-emotion, parenting stress, and child emotion regulation for parent/child dyads (*n* = 19)	Children were 39% White, 42% Latine, 6% other	Clinical—Parents of children with DD	None reported	None—focused on parental awareness of their own feelings about their own emotions (meta-emotion)
Zhang et al., 2015 [46]	Randomized controlled pilot study assessing impact of *Mindful Motherhood* on mindfulness, cortisol response, and stress with pregnant mothers in treatment (*n* = 34) and control (*n* = 31) groups	Women were 100% Black	Clinical—Women from low-income environments	None reported	Focused on individual mindfulness practice for pregnant mothers and cultivating capacity to be fully present more often with themselves and their children

^1^—Non-clinical samples—populations without a clinically diagnosed disorder or socioeconomic condition. ^2^—Cultural adaptions were operationalized to mean any changes to intervention delivery based on the cultural identity of the participants (such as modifications based on linguistic preferences, cultural heritage, spirituality, social needs, cultural values/priorities, etc.). ^3^—Attention-Deficit/Hyperactivity Disorder. ^4^—developmental delays. ^5^—Community-based participatory research (CBPR). ^6^—Intellectual Developmental Disorders. ^7^—randomized control trial. ^8^—Autism Spectrum Disorder. ^9^—Opioid Use Disorder. ^10^—Substance Use Disorder. ^11^—Fragile X Messenger Ribonucleoprotein gene. ^12^—Neonatal intensive care unit.

## Data Availability

The data that support the findings of this study are available from the corresponding author, J.C.W, upon reasonable request. Data sharing is not applicable to this article as no new data were created or analyzed in this study.

## Appendix A. Concept Table for Database Search Strategy

**Mindfulness** **[Concept A]**	**Parenting** **[Concept B]**	**Child Mental Health** **[Concept C]**
**Key Words**		
Mindfulness Relational mindfulness Meditation Mindful parenting Awareness Self-compassion Cognitive therapy Family-based intervention Parenting intervention	Parents Parenting Parenting behaviors Personal satisfaction Parent well-being Parenting stress Attachment Psychopathology Marital functioning Co-parenting Relationship quality Parent–child dyads Parent–child relations Interpersonal relations	Depression Anxiety Externalization Internalization Psychopathology Challenging behavior Adolescence Child internalizing Child externalizing Internal–external control
**MeSH**		
1. Mindfulness exp NT Self-compassion exp 2. Awareness exp 3. Meditation exp 4. Relaxation exp NT Rest exp	1. Family exp - Family relations - Family conflict - Intergenerational relations 2. Family characteristics exp - Marital status 3. Object Attachment exp	1. Mental disorders exp NT Trauma and Stressor related disorders 2. Emotions exp NT Depression exp NT Anxiety exp 3. Behavior exp 4. Psychopathology exp 5. Adolescent exp 6. Child exp 7. Infant exp
